# Distributed Energy IoT-Based Real-Time Virtual Energy Prosumer Business Model for Distributed Power Resource

**DOI:** 10.3390/s21134533

**Published:** 2021-07-01

**Authors:** Sanguk Park, Keonhee Cho, Seunghwan Kim, Guwon Yoon, Myeong-In Choi, Sangmin Park, Sehyun Park

**Affiliations:** 1 Department of Intelligent Energy and Industry, Chung-Ang University, Seoul 06974, Korea; pppssuu@cau.ac.kr (S.P.); motlover@cau.ac.kr (S.P.); 2 School of Electrical and Electronics Engineering, Chung-Ang University, Seoul 06974, Korea; thckwall@cau.ac.kr (K.C.); tkftn456@cau.ac.kr (S.K.); gw1206@cau.ac.kr (G.Y.); auddlscjswo@cau.ac.kr (M.-I.C.)

**Keywords:** distributed energy IoT (Internet of Things), intelligent energy management system (iEMS), virtual energy prosumer, energy trading, real-time energy sharing

## Abstract

Smart energy technologies, services, and business models are being developed to reduce energy consumption and emissions of CO_2_ and greenhouse gases and to build a sustainable environment. Renewable energy is being actively developed throughout the world, and many intelligent service models related to renewable energy are being proposed. One of the representative service models is the energy prosumer. Through energy trading, the demand for renewable energy and distributed power is efficiently managed, and insufficient energy is covered through energy transaction. Moreover, various incentives can be provided, such as reduced electricity bills. However, despite such a smart service, the energy prosumer model is difficult to expand into a practical business model for application in real life. This is because the production price of renewable energy is higher than that of the actual grid, and it is difficult to accurately set the selling price, restricting the formation of the actual market between sellers and consumers. To solve this problem, this paper proposes a small-scale energy transaction model between a seller and a buyer on a peer-to-peer (P2P) basis. This model employs a virtual prosumer management system that utilizes the existing grid and realizes the power system in real time without using an energy storage system (ESS). Thus, the profits of sellers and consumers of energy transactions are maximized with an improved return on investment (ROI), and an intelligent demand management system can be established.

## 1. Introduction

Currently, researchers throughout the world are working on the development of smart energy technologies, services, and business models to reduce energy consumption and emissions of CO_2_ and greenhouse gases. Thus, a sustainable environment can be built based on intelligent Information and Communication Technologies (ICT). The most common renewable energy is solar energy. Renewable energy systems based on solar energy and intelligent service models related to renewable energy are being developed. One of the representative service models is the energy prosumer [1,2,3,4]. An energy prosumer is a compound term for energy producers and consumers and refers to those who directly produce power through solar facilities in apartment complexes, university buildings, and industrial complexes [5].

Through an energy prosumer, various distributed and scattered power sources are efficiently managed and insufficient energy is covered through transactions, thereby providing various benefits for energy saving, such as reduced electricity bills [6,7,8]. The business of energy prosumers is related to changes in consumer behavior as a part of the new energy industry. The exchange of information between the supply and demand sides has enabled the establishment of a demand management system with an improved consumer demand response, energy saving, and efficiency improvement. In addition, by installing renewable energy sources directly, consumers can now build a self-sufficient energy management system that includes energy production and consumption [9,10,11,12]. Consumers throughout the world have already installed renewable energy facilities, particularly photovoltaic power generation facilities, and are trading surplus power in various ways [1,13,14]. In addition, the installation of solar panels by consumers is increasing rapidly because of the continuous decline in solar power generation costs and the continuous rise in electricity rates. Presently, it is cheaper for consumers to produce and consume electricity directly than to purchase it from a power company. Thus, it has been predicted that the centralized energy supply method centered on current large-scale facilities will change to a decentralized self-sufficient energy supply method [9].

However, despite the existence of such smart services, the energy trading model in Korea is difficult to expand into a practical business model for application in real life. This is because the production price of renewable energy is higher than that of the existing grid (EG), and it is difficult to accurately set the selling price, restricting the formation of a real market between sellers and consumers. To solve this problem, this paper proposes a small-scale energy trading model between a seller and a buyer on a peer-to-peer (P2P) basis using an EG. This model employs a virtual prosumer management system that utilizes the EG and enables power storage in real time without using an energy storage system (ESS). Because this model does not use an ESS, solar power generation costs can be reduced, and the resulting short return on investment (ROI) can maximize the profits of sellers and consumers of energy trading.

### 1.1. Current Energy Prosumer Problems

1. High ROI (first 10 years based on government subsidies): Renewable energy (solar power) facilities and ESS construction have a high ROI because the profits are not large compared to the high installation costs. Thus, they rely on government subsidies.
2. Difficulty in market formation: The production price of new and renewable energy is higher than that of the actual grid, and it is difficult to accurately set the sales price. Therefore, the actual market between sellers and consumers is not formed.

### 1.2. Solutions

1. Real-time energy trading: A virtual power bank (VPB)-based real-time virtual prosumer management system uses an existing power grid. It is composed of the virtual infrastructure of the EG (VPB), which does not include an ESS, and does not require a large cost to configure the ESS and its own energy trading grid. Thus, a large profit can be obtained at a low price.
2. Flexible cost setting: The prosumer market has a structure that allows both sellers and buyers to obtain profits by setting flexible sales/purchase prices for each situation and to provide the maximum benefit between electricity sellers and consumers rather than setting energy costs based on fixed electricity rates.

The remainder of this paper is organized as follows. Section 2 discusses relevant previous studies. Section 3 presents an overview of the proposed system, and Section 4 discusses the two business models. Section 5 demonstrates simulations conducted according to each situation by composing similar real scenarios and test beds and analyzing them. In Section 6, the conclusion of the study, the expected effects and future prospects for the final proposal system are presented. 

## 2. Related Works

This section examines the existing literature related to energy prosumers. Rathnayaka et al. [15] proposed an approach to assess each prosumer’s contribution to developing a sustainable prosumer and to find the subset of the most influential prosumers that act to promote long-term sustainability. In their work, they proposed an innovative methodology for evaluating and ranking prosumers to build an influential membership base. Moreover, they assessed the short- and long-term energy behaviors of prosumers based on multiple evaluations. Prosumers were ranked according to the criteria, and prosumers with higher ranking were considered to have a greater impact on strengthening long-term livelihoods. Ma et al. [16] proposed an energy management framework that focused on the energy management of microgrids, which were mainly composed of combined heat and power as well as photovoltaic (PV) prosumers. A multilateral energy management framework was proposed for the joint operation of prosumers along with internal price-based demand response. In particular, an optimization model based on the Stackelberg game was designed, with the micro grid operator acting as the leader and the PV prosumer as the follower. They studied the properties of the game and proved that the game has a unique Stackelberg balance. 

Luna et al. [17] proposed an energy management system to coordinate the operations of distributed household prosumers. They found that working with other prosumers in their surroundings could lead to better performance. They validated the proposed strategy by comparing the performance of an independent prosumer with that in cooperative mode. Shady et al. [18] considered the high penetration rate of prosumers equipped with rooftop solar power and electric vehicles, presenting a new approach to helping power distribution system operators to intelligently design community battery energy storage systems. Azar et al. [19] introduced a scalable framework that coordinates net load scheduling, sharing, and matching near grid-connected residential prosumers. Because prosumers are equipped with smart devices, solar panels, and battery energy storage systems, they utilize the flexibility of consumption, generation, and storage to exchange energy with surrounding prosumers by negotiating the amount and price of energy with an aggregator.

Cui et al. [20] proposed an energy sharing framework for a new prosumer microgrid with renewable energy, multiple storage devices, and load transfer. In the first phase, a robust dual-level energy sharing model is formulated to provide a robust energy sharing schedule for prosumers and retailers that overcomes the impact of market prices and uncertainties in renewable energy. In the second phase, an online optimization model is constructed such that each prosumer can continuously optimize the energy schedule every hour according to the state-of-the-art system, and the proposed punishment mechanism is built in for prosumers to adjust the previous energy-sharing schedule. He [21] presented an efficient P2P energy sharing framework for numerous community prosumers to reduce energy costs and promote renewable energy utilization. Specifically, a strategy for sharing energy between communities and a strategy for sharing energy within a community was proposed for daily and real-time energy management for prosumers. In the previous strategy, prosumers can share energy with all community peers, and community aggregators coordinate their energy sharing on behalf of their prosumers. Ghosh et al. [22] proposed a distributed algorithm that converges to the exchange price and demonstrated the selection of the price function in a day-ahead scenario using estimated demand from the history.

Chen et al. [23] proposed an interdisciplinary P2P energy sharing framework that considers both technical and sociological aspects. Prosumers act as followers through subjective loading strategies, while energy sharing providers (ESPs) act as leaders in dynamic pricing schemes based on prospect theory (PT) and probabilistic game theory. For the prosumer, the subjective utility model of the risk utility (RU) decided by PT is designed. For the ESP, a profit model for dynamic price is proposed, and a solution algorithm consisting of interpolation and curve fitting to obtain the RU function yields a Markov prosumer. It solves the “dimensional curse” and discreteness problems arising from the social nature of prosumers by incorporating them into the decision-making process and proposing a differential evolutionary algorithm to solve the game. This method is effective in terms of the social behavior of the prosumer, that is, conservatism in obtaining radical performance in defeat. 

Carli et al. [24] proposed a decentralized control strategy for the scheduling of electrical energy activities of a microgrid composed of smart homes connected to a distributor and exchanging renewable energy produced by individually owned distributed energy resources. Scarabaggio et al. [25] suggested a distributed demand-side management (DSM) approach for smart grids taking into account the uncertainty in wind power forecasting, defining an approach to cope with the uncertainty in wind power availability. Giraldo et al. [26] presented an energy management system (EMS) for single-phase or balanced three-phase microgrids via robust convex optimization, represented as a convex mixed-integer second-order cone programming model. Hosseini et al. [27] proposed a novel robust framework for the day-ahead energy scheduling of a residential microgrid comprising interconnected smart users, each with individual renewable energy sources, noncontrollable loads, energy and comfort-based controllable loads, and individual plug-in electric vehicles. These minimize the expected energy cost while satisfying device/comfort/contractual constraints, including feasibility constraints on energy transfer between users and the grid under renewable energy source generation and users’ demand uncertainties.

We investigated existing approaches related to energy prosumers. Based on this, the reasons in the difficulty for expanding the current energy trading models into a practical business model in real life were determined. First, the production price of renewable energy is higher than that of the actual grid. Second, the selling price is inaccurate. It is impossible to establish a real market between sellers and consumers. This paper proposes a virtual power bank (VPB)-based real-time virtual prosumer management system using an existing power grid. The model does not use an ESS and can thus reduce the cost of solar power generation and maximize the profits of prosumers and consumers of energy trading with a short ROI. Next, we analyze the novelties of the proposed paper and compare it with existing references in Table 1.

### 2.1. Analysis of the Existing References

As a result of reviewing existing papers, we found that an energy data prediction-based optimization system has already been implemented. The P2P-based prosumer system, efficient energy trading of PV in the microgrid, and an energy optimization plan based on game theory have also been well implemented. However, this study focuses on the price conditions that must be formed to trade solar energy in small spaces (homes, etc.). In other words, the study focuses on data-driven virtual energy management system research to maximize profits between prosumers and consumers at the lowest possible price without configuring independent local grids and ESSs, and to increase the efficiency of solar energy transactions. The remainder of this paper is organized as follows.

### 2.2. Novel Points of Proposed System from Existing References

(1) Data-driven Virtual Energy Management: Virtual energy trading is possible only through energy data analysis based on distributed energy IoT using a traditional grid without configuring an ESS-based independent local grid in the community space (apartment complex, etc.).
(2) Real-time energy demand management: Virtual energy trading is possible because of energy status monitoring and real-time energy offset by real-time energy data.
(3) Cost-effective energy trading system: It is possible to establish a cost-effective energy system by not installing the ESS and additional local grid.
(4) Energy cost saving: Prosumers sell higher than the existing solar energy transaction costs, and customers benefit from reducing electricity bills by mitigating the progressive electricity tax at home.

## 3. System Overview

**Figure 1:**
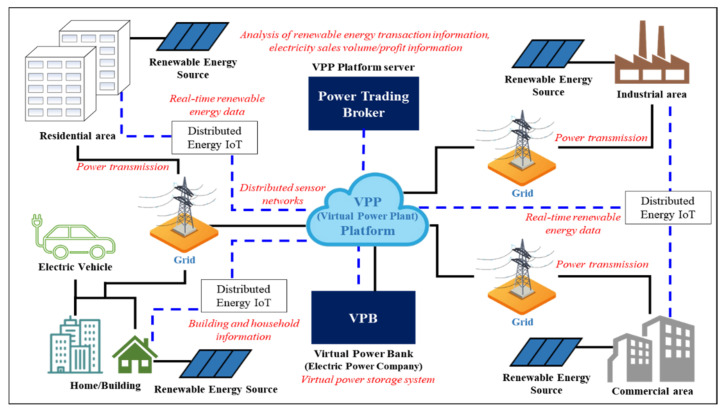
Schematic diagram of the total energy trading system.

Figure 1 shows a schematic of the total energy trading system. The system is configured with seven components: Power trading brokers, virtual power banks, residential areas, home/building areas, electric vehicles, industrial areas, and commercial areas. The power data are collected by the distributed energy IoT. Electric vehicles are one of the components of an emerging distributed system that constitutes the energy ecosystem in cities. However, the demand for electric vehicles was not reflected in the simulation test in this study because the purpose of this study is to generate profits via “energy trading.” The increasing demand for electric vehicles reflects the enormous demand within the city, and a business model that can generate profits in terms of “energy trading” must be further developed. However, first, whether there are any benefits to be gained from the role of electric vehicles in “energy trading” must be considered. 

There is a “progressive tax” for household electricity bills in Korea, which can provide the driving force for energy trading through renewable energy. To avoid the progressive tax, buyers purchase electricity from other prosumers, which is produced from renewable energy instead of from the existing grid. Even if the purchased electricity price is greater than the price of the existing grid, the customers will ultimately receive significant benefits if the final electricity price is not affected by the progressive tax. 

The power data are collected by the distributed energy IoT. Virtual energy trading is possible because of the energy status monitoring and real-time energy offset by real-time energy data through distributed energy IoT. Basically, in this study, we collect energy data based on the distributed energy IoT. Distributed energy IoT is an IoT device installed in each new renewable energy platform (solar panel) to collect data on scattered renewable energy (solar panels). Data such as power charging status and discharge status are collected through power sensor information collected from each distributed solar panel, and intelligent services are provided through intelligent data analysis algorithms based on these energy data [24,25,26,27]. 

The following Box 1 and Box 2 show the description of the components, the types of data collected from the distributed energy IoT, and detailed energy trading methods.

Box 1 **The description of the technical components.**

- Power trading broker: VPP platform server for analysis of energy trading data.
- Virtual power bank: Virtual power storage system for energy trading.
- Distributed energy IoT: IoT based distributed sensor network system.
- Renewable energy source: 325 W solar panel.

Box 2 **The types of data collected from the distributed energy IoT.**

- Building and household information: Building management information, building area, scope and gross area, etc.
- Building energy demand and supply status data: Electric energy, etc.
- Real-time generation information of renewable energy sources: Renewable energy sources, capacity, real-time generation, etc.
- Renewable energy surplus energy transaction information, electricity sales volume/profit information

### 3.1. System Architecture

The representative energy trading method is implemented as follows.

1. Category 1–Net metering (NM) with electric power corporation: The energy prosumer business through NM with a power company is an approach in which the power company purchases the surplus power produced by the consumer through solar power generation and lowers electricity bills. The NM with the power company is performed to reduce the electric charge to be paid by the consumer by calculating the electric charge for the pure electricity quantity obtained by deducting the surplus electric power from the electric power received by the electric power company [28,29,30].
2. Category 2—Energy trading of surplus power in local grid (LG): In terms of the sale of surplus power produced by a prosumer, it is the same as the NM of the power company. However, by selling the surplus power to the consumer, sales revenue is obtained separately from the electricity bill. In this case, PT plays the role of a medium to buy and sell surplus power. If the transaction price for surplus power is set rather than the physical flow of surplus power, the focus is on the transaction settlement for the sales profit of the prosumer and payment for the purchase by a net consumer [15,17].
3. Category 3—Power trading between prosumers over the Internet: The power trading platform built through the Internet makes it easier to sell surplus power produced by energy prosumers directly between individuals. The amount of surplus electricity and transaction price are set directly on the Internet without going through an intermediary, and the transaction can be concluded if there is a customer. Even in this case, if a transaction is applied, mutual benefits can be obtained through settlement between the parties dealing in the transaction. That is, a transaction price that is lower than the electricity rate and higher than the solar power generation unit price is generated. The prosumer has a profit even after paying the fee, and the customer can make a transaction if the purchase price is lower than the electricity rate even after the fee is paid. Power trading between individuals through the Internet power trading platform is in its early stages, and the number of countries using it through the experimental stage is gradually increasing. It is expected that power trading in this manner will increase in the future [31].
4. Category 4—Energy trading of surplus power using a distributed resource broker market: For the sale of surplus power by consumers using the distributed resource brokerage market, the brokerage company collects small-scale distributed resources, trades them in the power wholesale market, and issues a new renewable energy supply certificate. It receives sales profit, which is a business method that consumers share with brokers. In this project, there are variations in profits due to variation in the wholesale market price of electric power and the transaction price of the renewable energy supply certificate. Therefore, it is likely to be selected if the benefits are greater compared to the expected returns of different business model methods [32,33,34].

**Figure 2:**
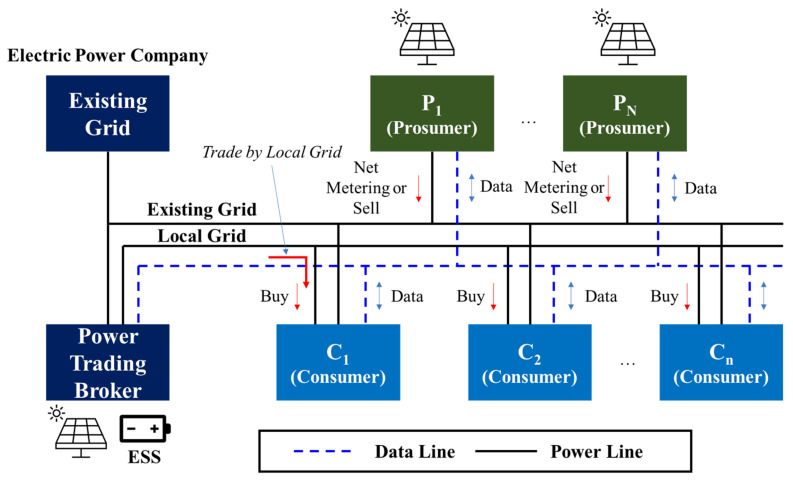
Schematic diagram of a typical energy trading system.

Figure 2 shows a schematic diagram of a typical energy trading system, showing the sales categories 1 and 2 of surplus electric power in the electric power institutions NM and LG. A typical energy trading system is largely composed of EG, $P_{N}$, $C_{n}$, and PTB. EG refers to the existing power grid and is connected to PTB-$P_{N}$-$C_{n}$. When excess power is generated, it can perform NM. NM is related to Category 1, which means that when surplus power is generated in a place where renewable energy is installed, it can be immediately sent back and sold to EG. The PTB acts as an intermediary to trade the power between $P_{N}$ and $C_{n}$. However, to trade electricity, LG is needed for power trading. Power trading between $P_{N}$ and $C_{n}$ is made through the LG. Box 3 shows the abbreviations.

Box 3 **Abbreviations.**

- PT (Power Trading): Selling surplus power remaining after self-exhaustion among energy produced by renewable energy to a second party
- EG (Existing Grid): Existing grid of electric power institution; KEPCO (Korea Electric Power Corporation)
- LG (Local Grid): Independent grid within the community for energy trading
- PTB (Power Trade Broker): A power broker for energy trading
- $P_{N}$ (Prosumer): Smallscale power seller
- $C_{n}$ (Customer): Small-scale personal power buyer
- NM (Net Metering): Sale of offset surplus power to the grid
- VPB (Virtual Power Bank): Store power using EG without building an additional independent grid or ESS within the community for energy trading.
- VG (Virtual Grid): A virtual connection chain made virtually based on data for energy trading, not a real grid.
- Virtual Power Plant (VPP) Platform: Energy data-based virtualization that uses energy data based on distributed energy IoT, monitors the storage of surplus power and transaction status between VPB and prosumer, and mediates virtually for transaction based on this power trading platform.
- $C_{NM}$: The cost when NM (KRW)
- $B_{NM}$ (NM benefit): Prosumer’s NM benefit (KRW)
- $B_{S}$ (Power trading benefit): Prosumer’s benefit by power trading (KRW)

**Figure 3:**
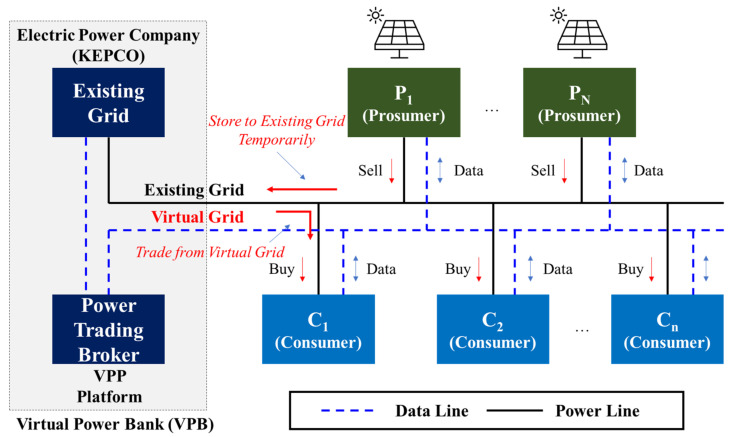
VPB-based power trading method.

Figure 3 presents the proposed VPB-based power trading method. The proposed power trading scheme generally involves three categories. The system comprises VPB (contains existing grid and VPP Platform), $P_{N}$, and $C_{n}$. VG represents the virtual power grid. This proposed system does not have LG or ESS. Although LG and ESS are essential for energy trading, they are eliminated owing to difficulties in construction and price issues, and VG is used as a complementary measure. This is to build VGs using EG. The VPP Platform monitors the energy trading status of each $P_{N}$ and $C_{n}$ based on the distributed energy IoT. Moreover, it mediates the transaction status virtually. 

First, $P_{N}$ immediately forms NM to EG when excess power is generated. However, this is a Virtual-NM concept that stores energy based on VPB, not the NM of Category 1. 

A virtual power bank (VPB) literally means virtual power storage, and the virtual power bank physically refers to the existing power grid, KEPCO (Korea Electric Power Corporation). Importantly, the existing power grid is used as virtual storage rather than physically building a large-capacity ESS. In terms of data, the VPB can be understood as a cloud-based system that utilizes a cloud server without building an individual server. The power of the produced renewable energy is sent to the existing grid and is not stored in a physical ESS (referred to as Net Metering (NM)). The existing power trading method is directly selling renewable energy to an electric power corporation to make a profit. However, the VPB-based power sales proposed in this paper do not sell power directly to the existing grid but temporarily store power in the VPB to sell it to other customers who need power (affected by progressive tax). The electricity produced is sent to the existing KEPCO grid, but not sold to KEPCO, and the power is owned by the prosumer, who produces it with renewable energy. In addition, because it uses the KEPCO grid infrastructure, a usage fee must be paid for using a certain amount of infrastructure. In this study, the virtual power bank is defined as a method of trading electricity with prosumers and customers.

In other words, energy is only stored in the VPB and not sold. The stored power is transacted with a customer ($C_{n}$), and the customer uses the power in EG only as much as the power stored by $P_{N}$. All these conditions are governed and managed by the VPP Platform. For the power trading market to operate actively, the prosumer and the customer must receive a greater profit than the current electricity bill. In other words, from the prosumer’s perspective, the power sales benefit ($B_{S}$) should be greater than the power offset benefit ($B_{NM}$) (1), and the consumer should have greater benefits when purchasing power from a prosumer than when the same amount is purchased from the electric power institution.

(1) $$B_{NM}\leq \;B_{S}$$

(2) The customer’s power trading and the cost paid by the electricity bill ≤ the customer’s existing electricity rate

### 3.2. System Configuration

**Figure 4:**
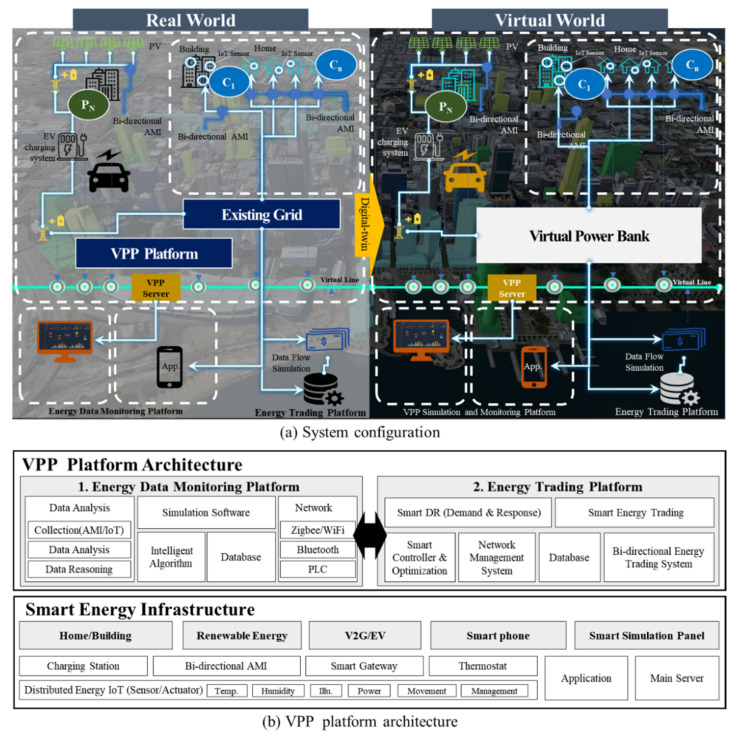
System configuration and architecture.

This thesis is based on three technologies: Energy data collection technology, energy data analysis technology, and energy trading technology (Figure 4). Energy data collection technology collects the status of renewable energy production and household energy consumption data in real-time through a distributed energy IoT (bidirectional advanced metering infrastructure (AMI)) installed in the home power infrastructure). It collects power data using an IoT-based metering sensor device. In the energy data analysis technology, the collected energy data are transmitted to the server, and data analysis and inference are performed to provide future intelligent energy transaction services via various intelligent data analysis algorithms The algorithms in the energy data monitoring platform are presented in Figure 4. The energy trading technology involves a local grid, which must be first configured for energy trading by the energy trading platform. It stores power generated from renewable energy in the ESS and trades energy via bidirectional energy transactions and bidirectional data transmissions (AMI).

**Figure 5:**
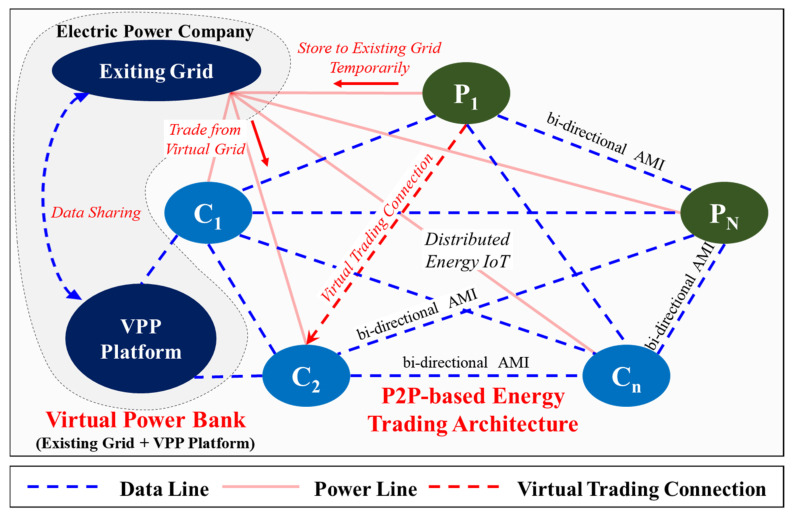
P2P-based proposed system architecture.

This paper proposes the concept of a VPB based on these three technologies. The proposed system implements a VPB-based real-time virtual prosumer management system using an existing power grid by applying the virtual power plant (VPP) concept without configuring a local grid for energy trading. This is excellent in terms of cost because it utilizes the existing power grid infrastructure without the additional configuration of the local grid and ESS by configuring a Virtual Trading Connection(VTC) as shown in Figure 5, i.e., it is very important to implement the data collection technology to the server to monitor and control energy transactions because it does not constitute the local grid. In other words, it is the core technology for monitoring and managing the overall status of energy transactions through the VPP platform technology, as shown in Figure 4 and Figure 5.

In addition, this paper proposes a small-scale energy transaction model between a seller and a buyer on a P2P basis. In other words, P2P is not a centralized method with autonomous interaction between peers for energy transactions without a server. However, in this paper, energy trading must take place at the platform level, and the management of energy data in the central data center is essential. Technically speaking, this is not a P2P-based energy transaction. 

The meaning of P2P transactions presented in this paper is not technical (without a server) but is used as a term to indicate a transaction in which residents in a residential area can freely participate in each other’s energy transactions. The system cannot operate if there is no server. The server is an essential element to compose the platform, and each prosumer’s transaction within the platform is based on P2P (social aspects). Figure 4 shows the flowchart of the system proposed in this study.

### 3.3. System Flow

**Figure 6:**
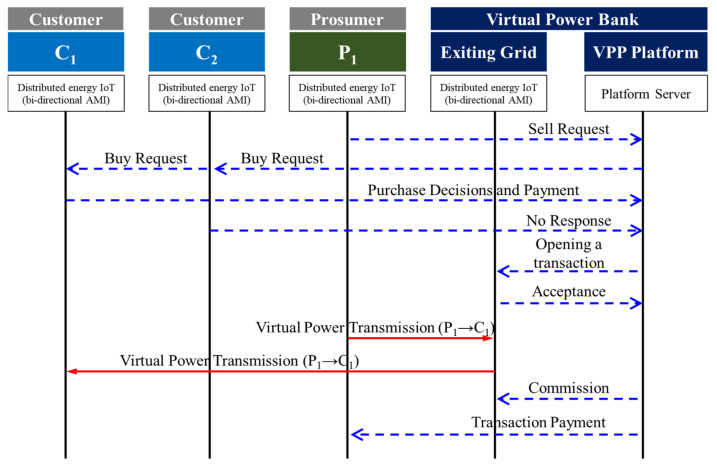
Flowchart of proposed system.

Figure 6 shows the flow of the proposed system, consisting of a VPP Platform, VPB, one prosumer, and two customers. First, Prosumer 1 requests sales to the VPP Platform to sell the surplus power of the solar power it produces. When requesting a sale, the VPP Platform delivers real-time information on the amount of electricity produced by the prosumer to the customer, and Customers 1 and 2 monitor the generated electricity to decide whether to purchase electricity. When the purchase intention is revealed to the VPP Platform, a trading session is formed, and the VPB notifies that a trading session has been opened. After confirming that the transaction session has been established, the VPB receives power from the prosumer. At this time, the power is received directly from the prosumer. However, this power is virtually stored in the VPB, and the first NM is processed. Customer 1 is given the right to use the amount of power NM from the prosumer of the VPB, and Customer 1 uses this power. At this time, payment for the power transaction is made in the VPP Platform, and the commission (grid usage fee) and the power price are delivered to the VPB and the prosumer, respectively.

## 4. Business Model

This paper proposes a VPB-based real-time virtual prosumer management system using an existing power grid. The specific scenarios of the model are described. Box 4 shows the abbreviations.

Box 4 **Abbreviations.**

- Prosumer N (P_N_): Households that sell surplus power with solar panels
- Consumer n (C_n_): Households that want to purchase power savings to reduce the progressive tax due to high power consumption
- Rn (Number of solar panels): Number of solar panels installed in the prosumer
- ROI (Return on Investment): Payback period for prosumer with solar power facilities (Year)

### 4.1. Scenario 1: Small-Scale Energy Trading Model in an Apartment Complex

**Figure 7:**
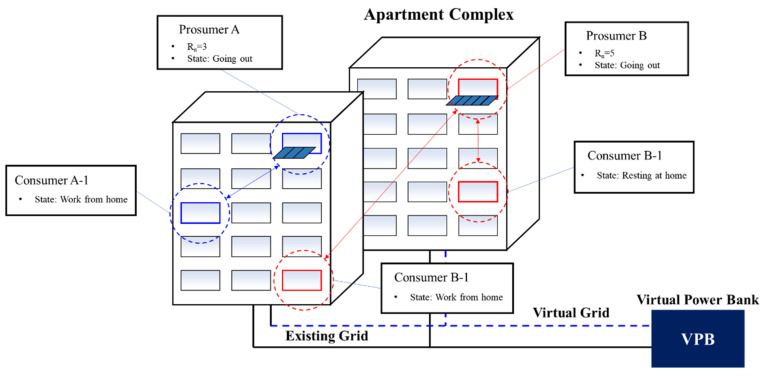
Small-scale energy trading model in an apartment complex.

- It is impossible to install a large-capacity PV in the apartment complex (Figure 7).
- There is no significant benefit to building a local grid in apartment complexes.
- It is dangerous to install ESS in an apartment complex, and ROI cannot be satisfied because it handles a small amount of PV even if it relies on government subsidies.
- Power loss is expected in the apartment complex during power transactions because the local grid is distant from the power trading company (intermediary).
- Therefore, to trade energy between prosumers in an apartment complex, a VPB-based real-time virtual prosumer management system using existing power grids is required.

### 4.2. Scenario 2: Surplus Electricity Trading Model Caused by Public Institution Closure Due to Pandemic

**Figure 8:**
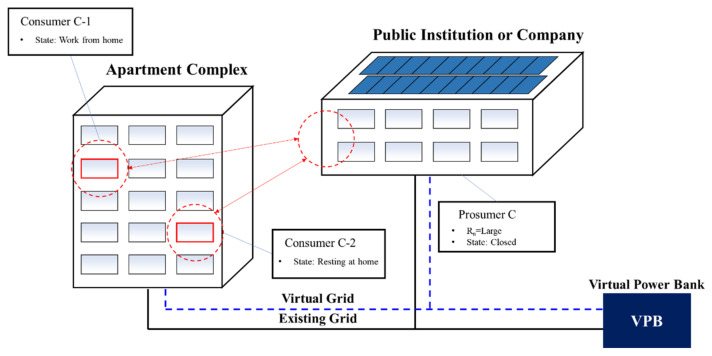
Surplus electricity trading model caused by public institution closure due to pandemic.

- Increasing number of telecommuters due to pandemic [35] (Figure 8).
- Most PVs are installed in buildings, such as public institutions. The energy consumption of public institutions is decreasing due to the increase in the number of telecommuters, and the energy consumption of apartment complexes is increasing.
- Most of the power produced at workplaces is lost because PV produces the most energy from 9:00 to 18:00 h during the daytime.
- The increase in telecommuting also increases the electricity demand for apartments between 9:00 and 18:00 h.
- For this, a plan is needed to mitigate the progressive tax through energy trading.
- VPB-based energy trading is required because it incurs a high cost to build a local grid in an apartment complex.

## 5. Simulation

The simulation test involved four types of households living in an apartment complex, each having four family members as follows.

1. Outgoing households: The household is empty as the entire family is on vacation or overseas business trip.
2. Households who are working from home: Family members are unable to go to work owing to a pandemic and are working from home.
3. Working households: The entire family is out in the afternoon because of work.
4. Nonworking households: Family members stay at home all day owing to holidays and are temporarily not working.

### 5.1. Typical Home Devices Used in One Household

Table 2 shows a list of devices (calculated based on power consumption) that a household typically uses in an apartment.

In this simulation, prosumer and customer were selected as follows.

- Power prosumer: Prosumer 1 (outgoing households): Households that have solar panels installed and have an empty house due to vacation or overseas business trips and generate excess power.
- Electricity customer 1: Customer 1 (households working from home): Households that are not able to go to work due to a pandemic and are working at home. Households are affected by a progressive tax as electricity use increases during the afternoon (working time: 09:00~18:00 h).
- Power purchaser 2: Customer 2 (nonworking households): Households affected by progressive tax due to high power consumption during the afternoon hours (9–18:00) because they do not work and stay at home all day.

Only Prosumer 1 is configured as a scenario for the following reasons. First, this study focuses on energy trading in a small space. Small-scale transactions reflect the benefits that can be obtained through small-scale electricity transactions by installing approximately three to five solar panels per household. Electricity trading is only possible when there is a surplus of electricity. Three to five solar panels can only produce approximately 2.944–4.906 kWh per day. Electricity at this capacity can be sold only when the prosumer is not at home. In other words, when a prosumer goes on a trip, the electricity generated from renewable energy in the house will be wasted. In this case, both the prosumer and customers can benefit from energy trading. However, if the prosumer is in the house, it would be prudent to consume the power generated, for personal use to achieve the highest profit, rather than trading energy. The priority is to solve the progressive tax from prosumers themselves. Therefore, the scenario model proposed in this paper is only applicable to Prosumer 1.

### 5.2. Actual Energy Consumption per Household 

The following shows the real-time energy production and consumption status for Prosumer 1 (Rn = 5). The solar panel used in the simulation had a solar efficiency of 325 W. The power produced by the solar panel was calculated based on the average value for the season, and the utilization rate data by time period was calculated based on the specifications of the solar panel installed in Jeju, Korea [36].

**Figure 9:**
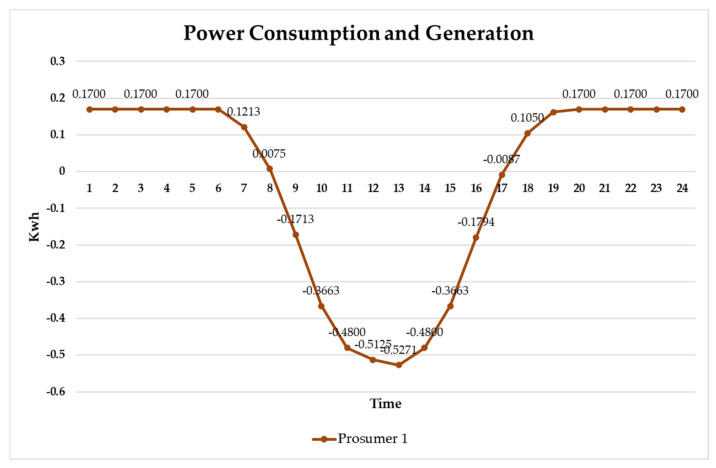
Real-time energy production and consumption status for Prosumer 1 (Rn = 5).

**Figure 10:**
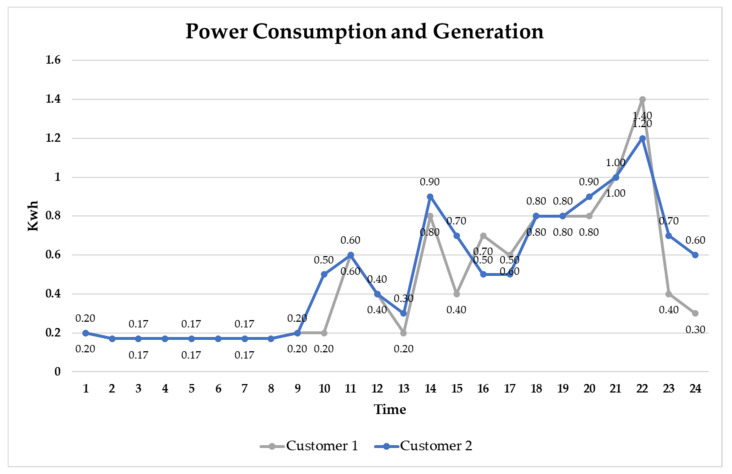
Consumption patterns of Customers 1 and 2.

Prosumer 1 is a household that has solar panels installed and generates surplus power because the entire family is on vacation or overseas business trips. Therefore, power is required for only devices (water purifiers, refrigerators, routers, wall pads, etc.) that use electricity continuously, and standby power is consumed, while surplus power is generated in 9–18 h (Figure 9). Figure 10 shows the real-time energy consumption status for Customer 1 (household working from home) and Customer 2 (nonworking household).

Focusing on the consumption patterns of Customers 1 and 2, there is a considerable power consumption between 9:00 and 18:00 h compared to working households because of the large amount of time spent at home due to telecommuting. This is the same time period in which Prosumer 1 produces power. Therefore, if the customer purchases the surplus power of Prosumer 1 in real time, the electricity consumed by working from home can be covered, thereby reducing electricity bills against progressive taxes.

### 5.3. Prosumer’s Optimal Number of Solar Panels for Each Customer

**Figure 11:**
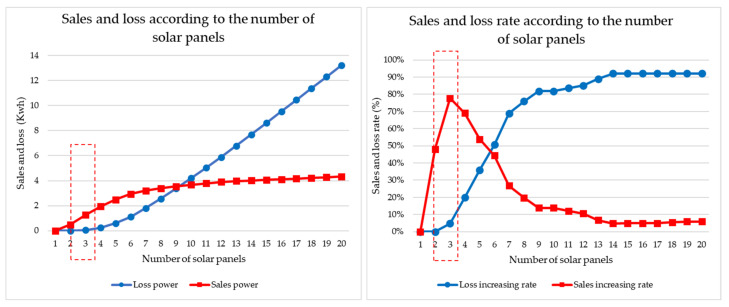
Prosumer’s optimal number of solar panels for each customer traded by Prosumer 1 and Customer 1.

We conducted a simulation of the optimal number of solar panels according to the number of targets traded by a prosumer. Simulations were conducted based on transactions between Prosumer 1 and Customer 1 and between Prosumer 1 and Customers 1 and 2. Figure 11 shows the results of a model traded by Prosumer 1-Customer 1 and a guide for setting the optimal amount of solar power when there is one power prosumer and one power customer. The graph on the left shows the power sales amount and surplus power loss value according to the number of solar panels of Prosumer 1. The graph on the right shows the increase in power sales and surplus power loss according to the amount of solar power obtained by differentiating this. As can be seen from the graph, the number of solar panels was selected at the point where the sales volume was the highest and the loss value was the minimum, and the number of solar panels with the minimum power loss was selected by limiting the number of apartment complexes. The number of panels was confirmed to be three (Rn = 3).

**Figure 12:**
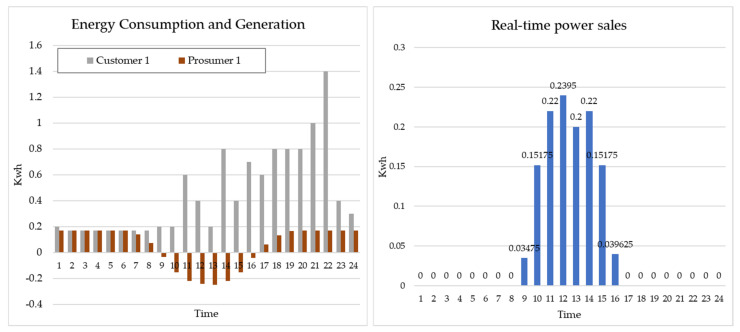
Real-time status of Prosumer 1 and Customer 1.

Figure 12 shows the real-time status of the energy trade model of Prosumer 1-Customer 1.

**Figure 13:**
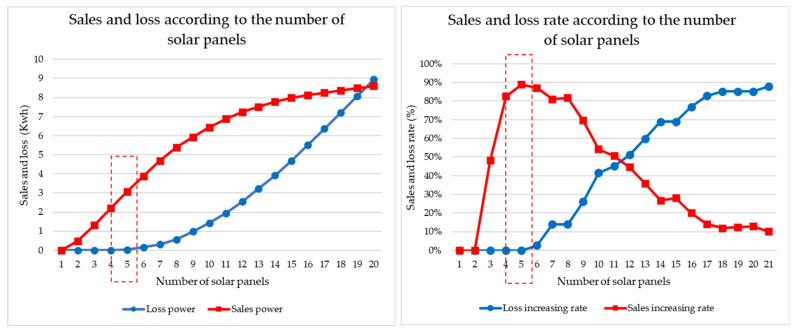
Prosumer’s optimal number of solar panels for each customer traded by Prosumer 1–Customer 1 and 2.

Figure 13 shows the results of a model of energy traded by Prosumer 1-Customer 1 and 2 and a guide for setting the optimal amount of solar power when there is one power prosumer and two power customers. The graph on the left shows the power sales amount and surplus power loss value according to the number of solar panels of Prosumer 1. The graph on the right shows the increase in power sales and surplus power loss according to the amount of solar power obtained by differentiating this. As can be seen from the graph, the number of solar panels was selected at the point where the sales volume was the highest and the loss value was the minimum, and the number of solar panels with the minimum power loss was selected by limiting the number of apartment complexes. Figure 11 shows that the number of panels was confirmed to be five (Rn = 5).

**Figure 14:**
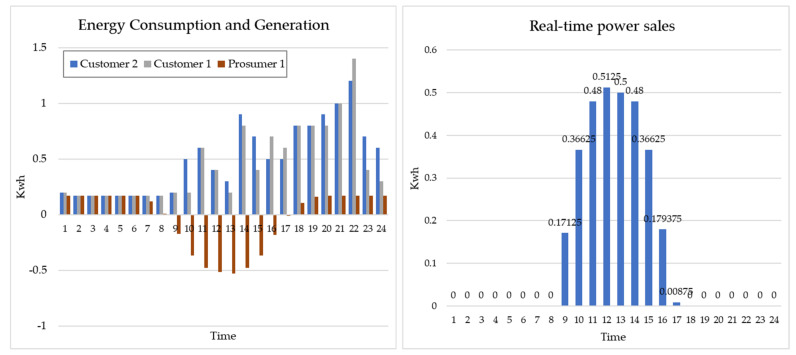
Real-time status of Prosumer 1-Customer 1 and 2.

Figure 14 shows the real-time status of the energy trade model of Prosumer 1-Customer 1 and 2.

### 5.4. Benefit and ROI Analysis for Optimal Prosumer Trading

In Section 3, we mentioned that the prosumer should have a higher $B_{S}$ than $B_{NM}$ (1) [9], and the consumer should have greater benefits when purchasing power from a prosumer than when the entire amount of power is purchased from an electric power institution (2). The following is a detailed description. Box 5 shows the abbreviations.

Box 5 **Abbreviations.**

- $B_{NM}$ (NM benefit): Prosumer’s NM benefit (KRW)
- $B_{S}$ (Power trading benefit): Prosumer’s benefit by power trading (KRW)
- C(x): Electricity charge paid to electric power institution for the power consumption x (KRW)
- $T_{p}$: Total amount consumed by the prosumer (kWh)
- $R_{p}$: Power received from electric power institution (kWh)
- $S_{p}$: The amount of electricity consumed by the prosumer (kWh)
- $T_{C}$: Total power used by customer (kWh)
- Z: Power purchased by customer (kWh)
- γ (Transaction ratio index): A coefficient to shorten the payback period of solar installation costs by multiplying the selling price of a certain ratio or higher to a prosumer who has installed solar panels rather than a consumer who does not have solar panels installed
- ε (Transaction profit index): A coefficient for the difference in profit between prosumer and customer

**Figure 15:**
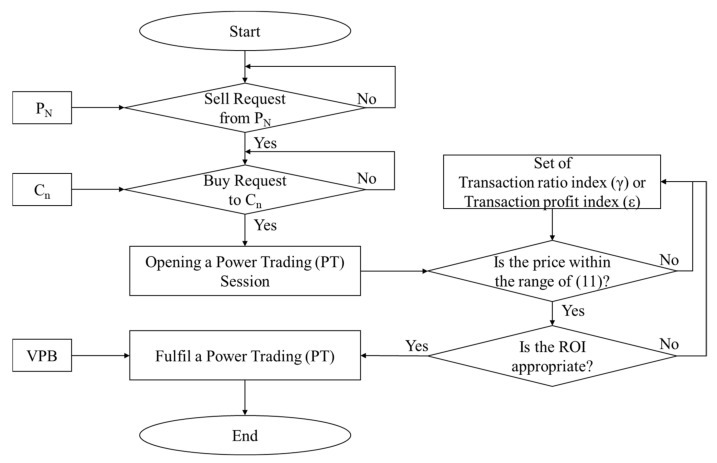
Algorithm of the proposed system.

Figure 15 shows the proposed system algorithm for VPB-based real-time virtual prosumer management system. Prosumer 1 requests sales to the VPP Platform to sell the surplus power of the solar power it produces. The VPP Platform opens a power trading session when it receives a buy request from a customer. From this point, the prosumer and the customer can trade energy, and the transaction price is determined by the change in the transaction ratio index (γ) or transaction profit index (ε) within the range of Equation (11). When the transaction price satisfies the optimum ROI and Equation (11), the transaction begins, and real-time transactions are made based on the VPB.

(3) $$B_{S}=C\left(T_{P}\right)-C\left(R_{P}\right)+P\cdot \left(Y-S_{P}\right)$$

(4) $$B_{NM}=C\left(T_{P}\right)-C\left(R_{P}-\left(Y-S_{P}\right)\right)$$

$$C\left(T_{P}\right)-C\left(R_{P}-\left(Y-S_{P}\right)\right)\;\leq \;C\left(T_{P}\right)-C\left(R_{P}\right)+P\cdot \left(Y-S_{P}\right)$$

(5) $$\frac{C\left(R_{P}\right)-C\left(R_{P}-\left(Y-S_{P}\right)\right)}{Y-S_{P}}\;\leq \;P$$

C(x) represents the electricity charge paid to the electric power institution for the power consumption x. $T_{P}$ represents the total amount (kwh, month) consumed by the prosumer, and $R_{P}$ represents the power received from the electric power institution (EG). $S_{P}$ is the amount of power consumed by the prosumer. $B_{S}$ is derived using Equation (3) and represents the sales of the surplus power remaining after generating electricity ($Y-S_{P}$, Y) is the total power generated by solar power), excluding the electricity bill received from the EG for the total power consumed by the prosumer. Combined prices lead to a profit in electricity sales.

$B_{NM}$ can be derived as shown in Equation (4). Subtracting the total power charge paid by NM of the surplus power, generated from solar power, from the charge for the total power consumption $T_{P}$, $B_{NM}$ is obtained. Therefore, the price of electricity traded in the prosumer market is derived using Equation (5).

(6) $$\gamma \frac{C\left(R_{P}\right)-C\left(R_{P}-\left(Y-S_{P}\right)\right)}{Y-S_{P}}\;\leq \;P$$

Equation (6) indicates that the profit in power trading should be greater than the profit in NM for the generated surplus power to the electric power institution. In addition, it shows the equation multiplied by γ, set for the recovery of solar panel installation cost of the prosumer who installed solar panels. The reason for multiplying γ was to shorten the payback period of solar installation cost by multiplying the selling price of a certain ratio or higher for a prosumer who installed solar panels rather than a consumer who did not install solar panels.

(7) $$\mathrm{Customer}’s\;\mathrm{existing}\;\mathrm{electricity}\;\mathrm{bill}=C(T_{C})$$

(8) $$\mathrm{Expenses}\;\mathrm{paid}\;\mathrm{by}\;\mathrm{customers}\;\mathrm{in}\;\mathrm{power}\;\mathrm{trading}\;\mathrm{and}\;\mathrm{electricity}\;\mathrm{bills}=C(T_{C}-Z)+P\cdot Z$$

Equations (7) and (8) describe calculations on the customer’s side. If the customer’s existing electricity bill is given by Equation (7), then the cost that the customer pays for power trading and electricity bills can be expressed as Equation (8). If the total power used by the customer is $T_{C}$ and the purchased power is Z, then the customer’s electricity rate should be less than the price before purchasing the existing electricity. Thus, the following Equations (9) and (10) can be derived.

(9) $$C\left(T_{C}-Z\right)+P\cdot Z\leq C\left(T_{C}\right)$$

(10) $$P\leq \;\frac{C\left(T_{C}\right)-C\left(T_{C}-Z\right)}{Z}$$

(11) $$\gamma \frac{C\left(R_{P}\right)-C\left(R_{P}-\left(Y-S_{P}\right)\right)}{Y-S_{P}}\;\leq \;P\;\leq \;\frac{C\left(T_{C}\right)-C\left(T_{C}-Z\right)}{Z}$$

Equation (11) can be obtained using Equations (6) and (10). In other words, for energy trading to be actively carried out, the energy trading price P must be in the range of Equation (11).

Table 3 and Table 4 show the profits and ROI from the perspective of prosumers and customers when a transaction is conducted based on simulated data based on the Prosumer 1-Customer 1 transaction model (Model 1) and Prosumer 1-Customers 1 and 2 transaction model (Model 2).

Table 4 shows that the profits and ROI that the prosumer and the customer can obtain when the VPB facility usage fee is 5%, and the difference in cost between Prosumer 1 and Customers 1 and 2 is set such that the prosumer can receive a profit of 1.7-times more than the customer (ε = 1.7).

**Figure 16:**
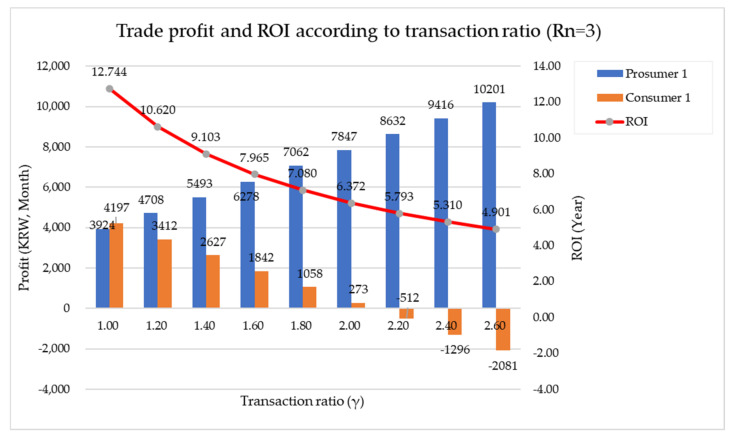
Transaction profit and ROI of Model 1 according to the change in γ.

**Figure 17:**
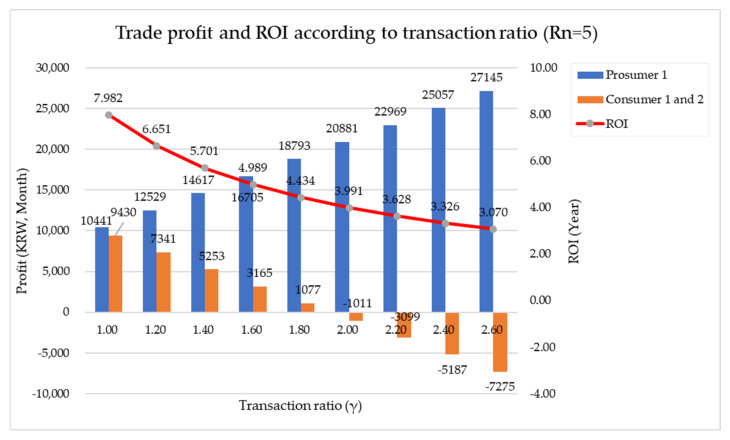
Transaction profit and ROI of Model 2 according to the change in γ.

In this study, the indicators suggested for price setting are γ and ε. As previously suggested, γ is a coefficient for shortening the payback period of solar panel installation costs by prosumers who have installed solar panels rather than consumers who have not installed solar panels. This is a coefficient that sets the selling price so that the prosumer can set the selling price P such that the prosumer can receive a higher profit than the customer (due to the payback period). The larger this value, the greater the profit for the prosumer. However, the transaction price P is the same for the prosumer or customer. In other words, there should be a difference in profits between the prosumer and the customer in terms of net profits from electricity transactions, not in terms of selling prices, and the coefficient for this is called ε. In this study, ε was set as 1.7. Figure 16 and Figure 17 show the change in transaction profit and ROI of Model 1 and Model 2, respectively, according to the change in γ.

**Figure 18:**
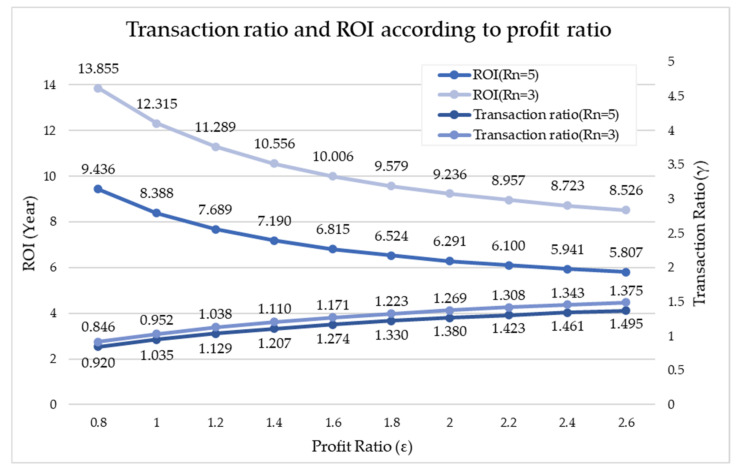
Change in γ and ROI according to the change in ε.

Figure 18 shows the change in γ and ROI according to the change in ε. It was found that as ε increased, γ gradually increased and ROI decreased. Comprehensively, when Rn = 3, for Prosumer 1 and Consumer 1 to obtain a profit, γ must be between 1 and 2, and when ε = 1.7, γ is 1.303. When Rn = 5, for Prosumer 1 and Consumers 1 and 2 to benefit, γ must be between 1 and 1.8, and at ε = 1.7, γ is 1.198. The following section presents the overall energy trading guidelines through this paper.

### 5.5. Guidelines for Small-Scale Power Trading

- The prosumer can sell power only when surplus power is produced by renewable energy.
- The prosumer obtains the maximum profit from self-consumption rather than selling it when the electricity produced by renewable energy is less than the consumed electricity.
- The prosumer can sell the surplus power when the power produced by renewable energy exceeds the consumed power.
- If the prosumer trades based on this proposed model when the electricity produced by renewable energy is more than the consumed electricity, higher profits can be achieved in small-scale electricity transactions than NM for the surplus electricity to EG.
- When the prosumer is not present, surplus power in the residence can be sold.
- The selling price should have a higher sales profit when sold to other customers (than the sales profit at NM with electric power institution), and the purchase price should be less than the electricity bill amount (including progressive tax) reduced by the customer’s electricity transaction. (Equation (11)).
- ESS is practically unnecessary for small power transactions if real-time transactions are possible because there is a capacity limit when installing an ESS.

## 6. Conclusions and Future Perspectives

In this paper, a business model for activating energy prosumers is presented. Despite the existence of smart energy trading services, the energy trading model is difficult to expand into a practical business model for application in real life. This is because the production price of new and renewable energy is higher than that of the actual grid, and it is difficult to accurately set the selling price, restricting the formation of a real market between the prosumer and the customer. To solve this problem, this paper proposes a small-scale power trading model between the prosumer and customers based on P2P using an EG. This model enables real-time power transactions without using ESS and is a virtual prosumer management system utilizing an EG. Because this model does not use ESS, it is suggested that solar power generation costs can be reduced, and the short ROI can maximize the profits of energy trading prosumers and customers. Based on a simulation test and scenario analysis, this system has significant future possibilities for microgrids:

- *Data-driven virtual energy management:* Virtual energy trading is possible only through energy data analysis based on distributed energy IoT using a traditional grid without configuring an ESS-based independent local grid in the community space (apartment complex, etc.).
- *Real-time energy demand management:* Virtual energy trading is possible because of energy status monitoring and real-time energy offset by real-time energy data.
- *Cost-effective energy trading system:* It is possible to establish a cost-effective energy system by not installing the ESS and additional local grid.
- *Energy cost saving:* Prosumers sell higher than the existing solar energy transaction costs, and customers benefit from reducing electricity bills by mitigating the progressive electricity tax at home.

In this study, simulations were performed for four types of home situations in an apartment complex with installed small solar panels. In addition, the economic effect was calculated by considering the situation when the P2P-based prosumer energy transaction was carried out after installing three or five 325 W solar panels.

This study included a simulation test with one prosumer and two customers. The simulations in this study were first performed to analyze the benefits in terms of price for small-scale energy transactions (household units). To prove the effectiveness of this, the minimum unit was established (one prosumer, one customer and one prosumer, two customers). This test was conducted based on actual household energy consumption data, the theoretical production of renewable energy (325 W solar capacity, collected from the Jeju area), and transaction price (electricity bills in Korea).

In principle, the theoretical analysis should be preceded by n prosumers and m consumers. However, the purpose of this study was to analyze small units based on actual scenarios and case studies, and the simulation was conducted based on this. Because the simulation in this study was the simplest and most basic unit, it needs to be further expanded and advanced in the future. In addition, additional research on the changing future energy elements (electric vehicles) presented in Section 3 and theoretical modeling for intelligent energy trade between prosumers and customers should be conducted.

Various energy sources and consumption factors (solar power, wind power, electric vehicles, etc.) have not yet been considered, and more complex intelligent algorithms are required because of the linkage of these diversifying energy factors [24,25]. Various energy factors must be considered, including electric vehicles, and more complex and advanced technologies will be required to apply not only to general households (apartment complexes) but also to public institutions, buildings, communities, and industrial complexes. This study focused on fixed renewable energy sources. However, it is also necessary to consider continuously diversifying new and renewable energy sources (fixed energy: Wind power/hydropower; mobile energy: Small-scale storage, bidirectional electric vehicles, etc.) [25,27].

## Figures and Tables

**Table 1:** Analysis of novelties of the proposed paper compared with existing references.

	Reference	Publication	Description	Novelties
1	“A methodology to find influential prosumers in prosumer community groups”	2013	Find influential prosumers by multiple assess system.	Cost-effective energy trading system
2	“Energy management for joint operation of CHP (combined heat and power) and PV prosumers inside a grid-connected microgrid: A game theoretic approach”	2016	A multilateral energy management framework and Stackelberg game–based optimization model.	Cost-effective energy trading system
3	“Cooperative energy management for a cluster of households prosumers”	2016	Energy management system to coordinate the operations of distributed household prosumers.	Cost-effective energy trading system
4	“Optimal design of community battery energy storage systems with prosumers owning electric vehicles”	2017	High penetration rate of prosumers equipped with rooftop solar power and electric vehicles.	Real-time energy demand management
5	“A non-cooperative framework for coordinating a neighborhood of distributed prosumers”	2018	A scalable framework that coordinates net load scheduling, sharing, and matching prosumers	Real-time energy demand management
6	“A two-stage robust energy-sharing management for prosumer microgrid”	2018	An energy-sharing framework for a new prosumer microgrid.	Cost-effective energy trading system
7	“An efficient peer-to-peer energy-sharing framework for numerous community prosumers”	2019	An efficient P2P energy-sharing framework for numerous community prosumers.	Cost-effective energy trading system
8	“Strategic prosumers: How to set the prices in a tiered market?”	2018	A distributed algorithm that converges to the exchange price and the price function in a day-ahead scenario.	VPP-based data-driven energy prosumer
9	“Peer-to-peer energy-sharing in distribution networks with multiple sharing regions”	2020	P2P energy-sharing framework that considers both technical and sociological aspects.	Cost-effective energy trading system
10	“Decentralized control for residential energy management of smart users’ microgrids with renewable energy exchange”	2019	Decentralized control strategy for the scheduling of electrical energy activities of a smart homes.	VPP-based data-driven energy prosumer
11	“Distributed demand-side management with stochastic wind power forecasting”	2021	A distributed demand-side management approach for smart grids taking into account uncertainty in wind power forecasting.	VPP-based data-driven energy prosumer
12	“Microgrids energy management using robust convex programming”	2019	An energy management system for single-phase or balanced three-phase microgrids via robust convex optimization.	VPP-based data-driven energy prosumer
13	“Robust optimal energy management of a residential microgrid under uncertainties on demand and renewable power generation”	2021	A novel robust framework for the day-ahead energy scheduling of a residential microgrid.	VPP-based data-driven energy prosumer

**Table 2:** Typical home devices used in one household.

Classification	Power Consumption		Total Hours Used	Total
	Always Use (W)	Partial Use (W)	Use Time (H)	
Rice cooker	-	122/366 (Keep warm)	0.5/5	1891
Highlight	-	5300	0.5	2650
Washing machine	-	1840	1	1840
Laundry dryer	-	1950	1	1950
Computer	-	50	6	300
Light	-	100	6	600
Coffee machine	-	800	0.2	160
Oven	-	1750	0.2	350
Microwave	-	1700	0.2	340
Massage chair	-	200	0.5	100
Home theater	-	90	0.5	45
Cleaner	-	15	0.1	1.5
Water purifier	20	-	24	480
Refrigerator	23	-	24	552
Router	15	-	24	360
Bidet	50	1050		0
Wall pad	9.5	20	24	228

**Table 3:** Profits and ROI from the perspective of prosumers and customers based on simulated data of Prosumer 1 and Customer 1 (kWh, KRW).

Classification	Solar Efficiency	Amount	Production Power (Day)	Production Power (Month)	Surplus Power	Sales and Purchases	Remaining after Sale	NM	Total Power Consumption	Electricity Bill	Profit	Transaction Ratio Index (γ)	Transaction Amount	Commission	Actual transaction Amount	Profit from Transactions	Solar Panel Installation	Profit (Year)	ROI
Prosumer	0.325	3	2.944	88.305	39.170	37.721	1.448	4130	-	-	-	1.3	5381.68	269.08	5112.5	5112.593	600,000	61,351.11	9.78
Customer 1	-	-	-	-	-	37.721	-	-	291.979	42,680	8120	-	-	-	5112.5	3007.407	-	-	-

**Table 4:** The profits and ROI from the perspective of prosumers and customers based on simulated data of Prosumer 1 and Customers 1 and 2 (kWh, KRW).

Classification	Solar Efficiency	Amount	Production Power (Day)	Production Power (Month)	Surplus Power	Sales and Purchases	Remaining after Sale	NM	Total Power Consumption	Electricity Bill	Profit	Transaction Ratio Index (γ)	Transaction Amount	Commission	Actual Transaction Amount	Profit from Transactions	Solar Panel Installation	Profit (Year)	ROI
Prosumer	0.325	5	4.906	147.176	92.745	91.931	0.814	10,990	-	-	-	1.19	13,169.2	658.46	12,510	12,510.7	1,000,000	150,129	6.661
Customer 1	-	-	-	-	-	44.465	-	-	285.234	41,190	9610	-	-	-	6051.2	3558.76	-	-	-
Customer 2	-	-	-	-	-	47.465	-	-	312.234	46,950	10,260	-	-	-	6459.5	3800.5	-	-	-

## Data Availability

Not applicable.
